# Does Determination of Initial Cluster Centroids Improve the Performance of *K*-Means Clustering Algorithm? Comparison of Three Hybrid Methods by Genetic Algorithm, Minimum Spanning Tree, and Hierarchical Clustering in an Applied Study

**DOI:** 10.1155/2020/7636857

**Published:** 2020-08-01

**Authors:** Saeedeh Pourahmad, Atefeh Basirat, Amir Rahimi, Marziyeh Doostfatemeh

**Affiliations:** ^1^Bioinformatics and Computational Biology Research Center, Shiraz University of Medical Sciences, Shiraz, Iran; ^2^Biostatistics Department, Medical School, Shiraz University of Medical Sciences, Shiraz, Iran; ^3^Department of Molecular Medicine, School of Advanced Medical Sciences and Technologies, Shiraz University of Medical Sciences, Shiraz, Iran

## Abstract

Random selection of initial centroids (centers) for clusters is a fundamental defect in *K*-means clustering algorithm as the algorithm's performance depends on initial centroids and may end up in local optimizations. Various hybrid methods have been introduced to resolve this defect in *K*-means clustering algorithm. As regards, there are no comparative studies comparing these methods in various aspects, the present paper compared three hybrid methods with *K*-means clustering algorithm using concepts of genetic algorithm, minimum spanning tree, and hierarchical clustering method. Although these three hybrid methods have received more attention in previous researches, fewer studies have compared their results. Hence, seven quantitative datasets with different characteristics in terms of sample size, number of features, and number of different classes are utilized in present study. Eleven indices of external and internal evaluating index were also considered for comparing the methods. Data indicated that the hybrid methods resulted in higher convergence rate in obtaining the final solution than the ordinary *K*-means method. Furthermore, the hybrid method with hierarchical clustering algorithm converges to the optimal solution with less iteration than the other two hybrid methods. However, hybrid methods with minimal spanning trees and genetic algorithms may not always or often be more effective than the ordinary *K*-means method. Therefore, despite the computational complexity, these three hybrid methods have not led to much improvement in the *K*-means method. However, a simulation study is required to compare the methods and complete the conclusion.

## 1. Introduction

Clustering is a branch of unsupervised learning. This method is widely used as a first step to interpret the data. In this method, samples are divided into groups whose members are similar to each other [1]. A good clustering algorithm should be efficient, reliable, and capable to determine relevant clusters [2]. From the four famous crisp clustering branches including distribution-based, density-based, connection-based, and partition-based methods, EM (expectation-maximization) algorithm; DBScan (density-based spatial clustering of applications with noise), hierarchichal, and *K*-means clustering methods can be pointed out, respectively [1]. Of course, there are other categories in clustering methods such as fuzzy clustering algorithms (such as fuzzy *C*-means) which are not in the scope of the present research.

*K*-means clustering is an important and popular technique in data mining. This method is a partition-based clustering algorithm which works with randomly selected points as the initial centroids (centers) at first and then updates these centroids in an iteratively process until some convergence criteria were met. The simplicity of *K*-means clustering method makes it as a basic and popular method in different fields of research. The most important function of this method is that it works better when the clusters overlap [3]. This method also works with high-volume data. However, the more clusters there are, the more *K*-means may fail to find all clusters correctly [3]. In addition, the clusters created in this method are spherical and convex. Its performance also depends on the initial centroids of clusters and often ends in the local optimization [3, 4]. To solve these problems, different hybrid methods have been proposed [5–25]. Some of them try to solve *K*-means problems by different methods [5–10, 12–20], and some others use the simplicity of *K*-means method to improve the performance of other clustering methods [11, 21–24]. The present paper evaluated the results of three well-known hybrid *K*-means methods with minimum spanning tree (MST) [5], genetic algorithm (GA) [6], and hierarchical clustering [7] in different datasets. Genetic algorithm is a good option to solve the local optimization of *K*-means and will give a proper initial cluster center [25]. Clustering based on MST is known for deriving disordered boundaries and outlier detection [23]. The MST-based clustering techniques have widely been used for efficient clustering [23, 24]. The combination of partition-based and hierarchical clustering methods may also strengthen both approaches and discard their disadvantages [7].

Meanwhile, an important task in cluster analysis is evaluating the results of a clustering method or comparing it to another clustering result. Lots of different validity measures have been proposed in the literature [26]. Among these evaluating methods, we applied eleven validity indices (internal, external, and relative) to judge or compare the results of clustering methods. Therefore, the analysis was performed in two phases. In phase I, to investigate whether *K*-means is a proper clustering method for each dataset, EM, DBScan, hierarchical, and *K*-means clustering methods were applied at first. Then, three hybrid methods were tested on each data in phase II and compared with the results of phase I.

Accordingly, the organization of this paper is as follows. Ordinary *K*-means algorithm is briefly reviewed along with three hybrid methods in Section 2. Also, seven internet datasets utilized in present study are introduced there. On Section 3, four ordinary clustering algorithms including *K*-means, hierarchical, DBScan, and EM algorithms accompanied with three hybrid methods including MST-based, GA-based, and hierarchical-based *K*-means methods are applied on each dataset, and the results of eleven different external and internal evaluation indices are reported for comparison. Section 4 contains some discussion on comparing these methods.

## 2. Materials and Methods

### 2.1. Materials

All hybrid methods introduced with *K*-means algorithm in the present paper with different underlying theories help improve this method by eliminating the random selection defects of initial centroids in the *K*-means clustering method. The nature of these hybrid methods can be influenced by various factors such as number of variables (features) in the dataset, sample size, and even number of labels (number of classes) in the data and exhibit quite different results. Since these hybrid methods in a dataset have not been compared yet, seven web datasets with different features were used in order to investigate the performance of these methods in the present paper. The data consisted of three gene expression datasets relating to leukemia, prostate, and colon cancers, and they were considered as high-dimensional data with an expression of more than 20,000 genes and were downloaded from the Gene Expression Omnibus (GEO) database (Table 1). Another four datasets were also well-known standard and appropriate Internet data for clustering methods and have been used in most applied papers to measure the performance of clustering methods (for instances [5, 6]). These data are available to all researchers for scientific research in the UCI database (University of California Irvine (UCI): Center for Machine Learning and Intelligent System) (Table 1).

### 2.2. Methods

#### 2.2.1. *K*-Means Clustering Method

The basic idea in *K*-means clustering includes the definition of clusters in a way that total within-cluster variation is minimized. There are many algorithms for *K*-means clustering method. Mac-Queen algorithm was used in the present paper [27] that defined the total within-cluster variations as the sum of squares of the Euclidean distance between objects and centroids. Let *X* = {*x*_*i*_}, *i* = 1, 2, ⋯., *n* be the set of *nd*-dimensional observations (points) to be clustered into a set of *K* clusters, *C* = {*c*_*k*_, *k* = 1, 2, ⋯, *K*}. *K*-means algorithm finds a partition such that the squared error between the center (empirical mean) of a cluster and the points in the cluster is minimized. Let *μ*_*k*_ be the mean of cluster*c*_*k*_. The sum of squared error (SSE) between *μ*_*k*_ and the points in cluster *c*_*k*_ is defined as [28]

(1) $$\begin{array}{c} \text{SSE}\left(c_{k}\right)=\underset{x_{i}\in c_{k}}{\sum }{\left\|x_{i}-\mu_{k}\right\|}^{2} \end{array}$$

The goal of *K*-means is to minimize the sum of the squared error over all *K* clusters,

(2) $$\begin{array}{c} \text{SSE}\left(C\right)=\overset{K}{\underset{k=1}{\sum }}\underset{x_{i}\in c_{k}}{\sum }{\left\|x_{i}-\mu_{k}\right\|}^{2} \end{array}$$

**Figure 1:**
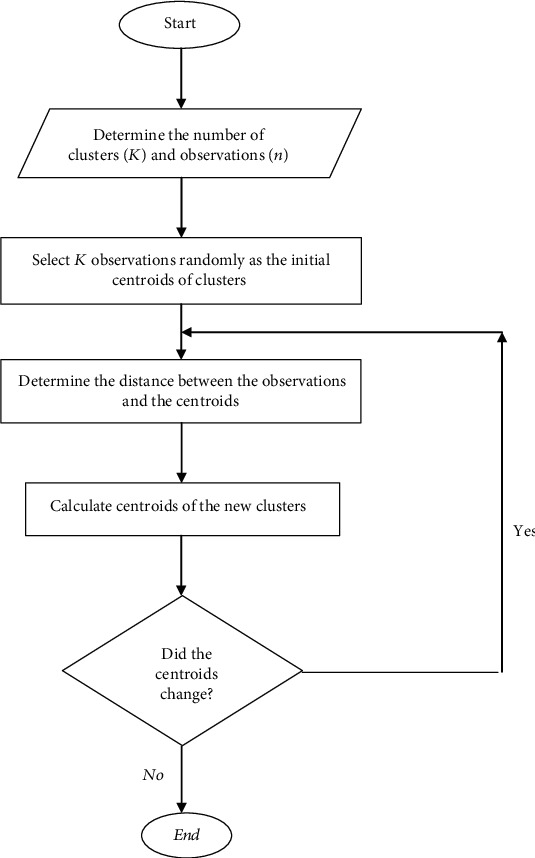
*K*-means clustering algorithm.

In general, the algorithmic steps of this method are summarized as follows (Figure 1):

1. Initial *K* cluster centroids are selected randomly from the observations
2. Distance between each observation and clusters' centroid is calculated and the observation is assigned to a cluster with minimal distance from the centroid of that cluster
3. Cluster centroids are updated by averaging the observations contained in each cluster
4. Distance between each observation and new centroids of clusters is recalculated and data are placed in new clusters based on the minimum distance to the centroids
5. Steps 3 and 4 are repeated until the centroids of clusters are not changed and the convergence occurs

#### 2.2.2. Combination of *K*-Means Clustering Algorithm with Minimum Spanning Tree Method

Minimum spanning trees (MSTs) have been applied in data mining, pattern recognition, and machine learning for a long time [3]. The MST-based clustering techniques usually lead to efficient clustering [23, 24]. Indeed, these hybrid clustering methods can identify clusters of arbitrary shape by removing inconsistent edges and detect clusters of heterogeneous nature. MST-based clustering algorithm was proposed by Zahn [23]. Since then, some studies have been conducted to improve it (such as [5, 23, 24]). MST is utilized as the preanalysis method to find the initial centroids for *K*-means algorithm [5].

In graph theory, a dataset can be shown by a complete graph *G*, so that number of vertices of graph indicates number of points in a dataset. The weight between two vertices is the Euclidean distance between points based on the features (variables) vector.

Tree is an undirected connected graph that does not contain any distance. The spanning tree is a subset of a complete weighted graph in a way that it has all features of a tree and also contains all vertices of a complete weighted graph. For a complete weighted graph, the minimum spanning tree has the least weight among all spanning trees of that graph. In present study, we followed the idea introduced by Yang et al. [5] and used MST for initializing the *K*-means clustering algorithm.

Accordingly, the MST-based *K*-means clustering algorithm applied in present study is as follows [5]:

(1) Number of points (*n* observations) and number of clusters (*K*) are entered as the input parameters
(2) MST is generated using Prim's algorithm
(3) The set *S* = {*s*_1_, *s*_2_, ⋯, *s*_*m*_}  is created containing the skeleton points from which the most edges pass (the number of edges from each point is known as degree). Member of *S* contains *m* points which admit some specific criteria (see ref. 6 for details) and are important candidates for cluster centroids at the first stage of *K*-means clustering
(4) Distances between any two skeleton points of *S* set are calculated (Equation (3))
  (3) $$\begin{array}{c} h\left(s_{i},c_{i}\right)=\left(v_{i}+v_{j}\right)\times d\left(s_{i},s_{j}\right) \end{array}$$
  where *v*_*i*_ and *v*_*j*_ are the degree of *s*_*i*_ and *s*_*j*_, respectively.
(5) The skeleton point *s*_*i*_ with the highest degree is selected and entered to the set of initial centroids denoted by *C* = {*s*_*i*_}
(6) The rest skeleton point *r*_*i*_  of *S* satisfying Equation (4) is added to set *C* = {*r*_*i*_} ∪ *C*

(4) $$\begin{array}{c} r_{i}=\max_{s_{i\in s}}\left(\min_{c_{i\in c}}\left(h\left(s_{i},c_{i}\right)\right)\right) \end{array}$$

Step 6 is repeated until the number of initial cluster centroids is equal to *K*.

**Figure 2:**
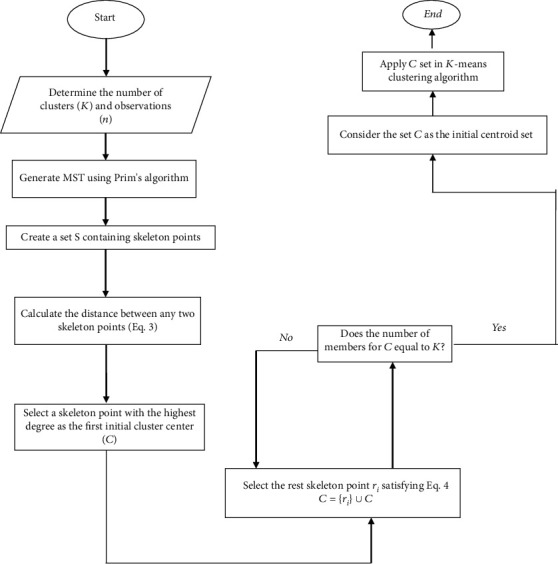
MST-based *K*-means clustering algorithm.

Figure 2 describes this process in a flowchart.

#### 2.2.3. Combination of *K*-Means Clustering with Genetic Algorithm

The genetic algorithms (GAs) in clustering analysis are usually used to determine the number of clusters automatically and to find initial centroids for *K*-means clustering [16]. Indeed, genetic algorithm is a good option to solve the local minimum problem of *K*-means [25]. Usually, the simplicity of the *K*-means algorithm and the ability of the genetic algorithm are combined to provide a GA-based clustering algorithm which has even attracted the attention of researchers in health sciences ( [17–21] for instances).

**Figure 3:**
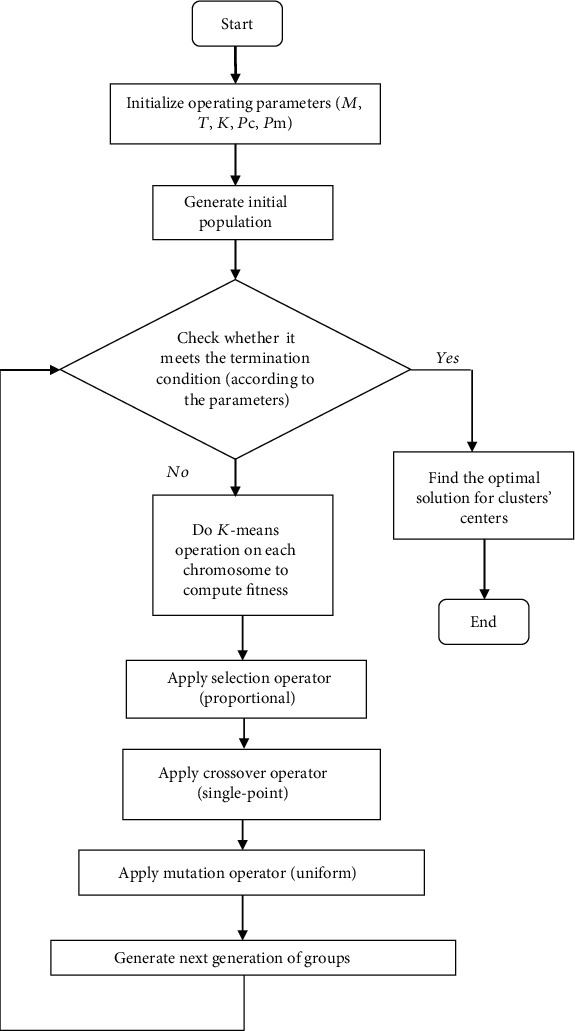
GA-based *K*-means clustering algoritm.

The genetic algorithm is inspired by the genetic science and Darwin's theory of evolution and is based on the survival of the superiors or natural selection. A common application of genetic algorithms is its use as an optimizer function. Inspired by the evolutionary process of nature, these algorithms solve problems. In other words, they create a population of beings like nature, and reach an optimal set or being by acting on this set. The hybrid method used in the present paper provided a hybrid version of the *K*-means algorithm with genetic algorithm that effectively solved the problem of random selection of initial centroids. Results of simulation tests confirmed this claim [11]. This algorithm preserved all important properties of the *K*-means method and is also stronger in data contains outlier. In general, the steps of GA-based *K*-means clustering algorithm are as follows (see ref. [6] for details):

1. The input parameters are determined including *M* initial population size (number of chromosomes) and *T* number of iterations (number of generations) and *K* number of clusters and operator rates *P*_*C*_, *P*_*m*_, ⋯
2. *M* chromosomes are randomly selected to generate the initial population where each chromosome is a set of initial centroids of clusters considering the notion that centroids of each chromosome should not be similarly selected
3. A target function is calculated for each chromosome. Based on the target function, the fitness value is calculated
4. Crossover, selection, and mutation operators are used to generate the next generation
5. If the number of produced generations is less than number of generations that is determined by user, it goes to stage 3 otherwise, it goes to stage 6
6. The amount of fit is calculated for the last generation per chromosome and compares the optimal amount of fit in this generation with the best fit obtained from previous generations and selects the largest one based on the estimator function
7. Finally, the initial centroids obtained from the best chromosome are used according to stage 2 as the initial centroids in the *K*-means clustering method (Figure 3)

#### 2.2.4. Combination of *K*-Means Clustering Algorithm with Hierarchical Clustering Method

The hierarchical method is the second most important crisp clustering method in microarray technology. In this method, clusters are formed by calculating the size of similarities or distance between each pair of elements [27]. The number of clusters is also determined by the users based on the height that the clusters merge. The weak point of hierarchical clustering is its termination, and the most important problem of *K*-means is its initiation [7]. Therefore, the combination of these two methods leads to a hybrid method with interesting characteristics. In present study, we apply agglomerative hierarchical clustering algorithm on a dataset at first to get initial information (initial centroids of clusters). Then *K*-means algorithm is applied.

Steps of hierarchical-based *K*-means method are summarized as follows [7]:

1. An agglomerative hierarchical clustering method is applied to data and the resulting tree is divided by *K* cluster
2. Centroid of each cluster (mean clusters) is calculated and set *C* is created
3. *K*-means algorithm is performed for the set *C* as the initial centroids obtained in step 2

**Figure 4:**
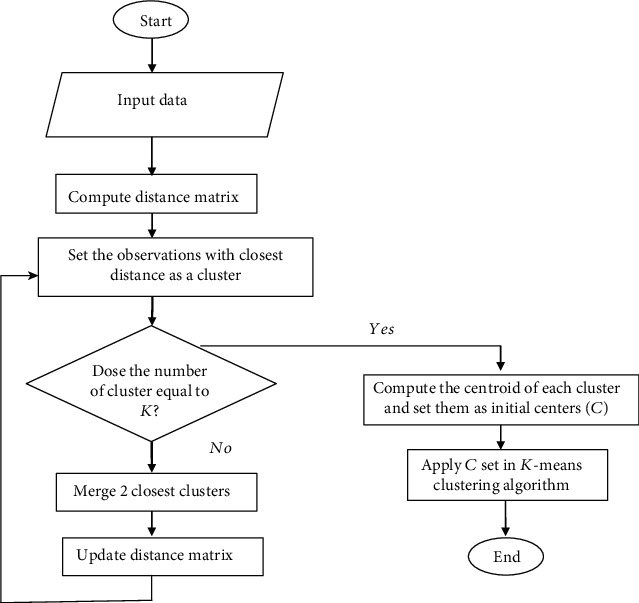
Hierarchical-based *K*-means clustering algoritm.

Figure 4 summarizes this algorithm through a flowchart.

#### 2.2.5. Validation of Clustering Methods

To evaluate the results of clustering algorithms, some cluster validation methods are used. These methods prevent the occurrence of random patterns in data and also allow the comparison of different clustering algorithms. A good validity measure should be invariant to the changes of sample size, cluster size, and number of clusters [26].

In general, clustering evaluation indices are classified into three categories: *internal*, *external*, and *relative*. Internal validity indices measure compactness, connectedness, and separation of each cluster while external validity indices measure matching the results of a clustering to the truth (if available) or another clustering method [26]. Relative validity methods in comparison are used to determine the optimal input parameter by changing the values such as the number of clusters in *K*-means for an instance and also comparing the clustering methods.

*Silhouette criterion* (Si), *Dunn index*, and the hybrid index *robustness*-*performance trade*-*off* (RPT) were applied in the present study for internal evaluation. External validity methods can be categorized into *pair*-*counting*, *information theoretic*, and *set matching measures*. *Pair*-*counting measures* (such as *rand index* (RI) and *adjusted rand index (ARI)* used in our research) are based on counting the pairs of objects in a dataset on which two different partitions agree or disagree. For instance, if two objects in one cluster in the first partition place also in the same cluster in the second partition, it is considered an agreement [26].

*Information theoretic indices* such as *mutual information* (MI) measures the information that two clustering methods share and *variation of information* (VI) as a simple linear explanation of MI is applied in the present study. *Set matching indices* such as *accuracy* (AC), *Fmeasure*, and *Haber*'*s Γ index* (HI) utilized here are based on pairing similar clusters in two partitions.

It should be noted that the optimal number of clusters in the present paper was determined by the majority rule and using three methods including the *average silhouette criteria*, *gap statistics*, and *elbow*; data were standardized prior to any clustering analysis.

## 3. Results

To compare the performance of three hybrid methods and ordinary *K*-means method, seven free downloadable datasets on Internet including “leukemia cancer,” “prostate cancer,” and “colon cancer” from GEO site and “haberman,” “iris,” “wine,” and “glass” datasets from UCI: Center for Machine Learning and Intelligent System were applied. Table 1 summarizes the description of these datasets.

To decrease the dimension of gene expression datasets and find the important genes (attributes), the result of the article by Ram et al. [29] was used. They selected a subset of three or four genes as the important ones by a feature selection method based on the random forest model. The clustering methods (ordinary or hybrid) were applied to the selected subsets for these four datasets.

It is necessary to mention that these datasets already contain some classes (labels). Ignoring these classes, we obtain the optimal number of clusters (among 2-15 clusters) for each dataset based on the majority rule according to the mean value of *silhouette*, *elbow* criterion, and *gap* statistics.

Then, the data analysis was organized in two phases:

### 3.1. Phase I

To investigate whether *K*-means is an appropriate clustering method for each dataset, four ordinary clustering methods including *K*-means, DBScan, hierarchical, and EM algorithms were applied on datasets at first. The mean value of *silhouette* and *RPT* criterion were then used to determine the best method for each data set (Table 2). The mean value of *silhouette* near to *one* and the high value of *RPT* reveal good clustering. Accordingly, *K*-means clustering method was the best method for just two out of seven data sets discussed in present study, leukemia and colon cancer datasets. Hierarchal clustering method was the best for prostate and haberman, the DBScan method was the best for iris and glass datasets and EM algorithm was the best method for the wine dataset.

### 3.2. Phase II

**Figure 5:**
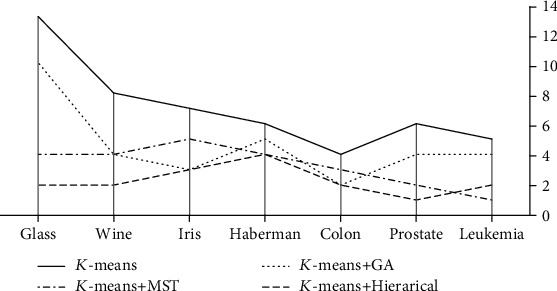
Number of iterations to converge for the hybrid methods in comparison with *K*-means method.

The hybrid *K*-means methods were then applied on each dataset, and the results were summarized in Table 3. The higher the value of these evaluation criteria, the better is the clustering algorithm; except for SSE and VI indices (their fewer values are desirable).  Figure 5 detects that all hybrid methods are faster in convergence than *K*-means method in terms of the number of iterations (the line belongs to *K*-means dominates the others).

Obviously, based on all evaluation criteria, one superior clustering method could not be achieved. But, depending on the purpose of the study, internal or external validity indices may be important. Therefore, according to internal validity indices, the MST-based clustering method was the best for all datasets except for the leukemia, wine, and glass datasets. For the former, GA-based and for the two latter, hierarchal-based methods are the best hybrid method (Table 3). However, the internal validity indices for the best hybrid method could not reach the values for the best ordinary clustering method determined in phase I (Tables 2 and 3), except for those two dat sets (leukemia and olon cancer) which *K*-means was the best ordinary method. According to external validity indices, MST-based clustering method for leukemia and haberman, GA-based method for prostate and hierarchal-based method for iris and glass were the best hybrid clustering methods. For colon cancer and wine datasets, all three hybrid methods have the same performance.

Totally, the hybrid methods could not greatly improve the performance of *K*-means clustering method in the present study. Meanwhile, although the results do not reveal any regular relationship between sample sizes, number of variables, and number of classes with the best hybrid method, but it seems hierarchal-based method works better in larger sample size with more variables (in wine and glass datasets).

## 4. Discussion

We have conducted a comparison study on three hybrid clustering methods which try to solve the random centroids problem in *K*-means clustering [5–7]. Seven existing datasets on Internet were applied to compare the methods. Eleven indices from different clusters' validation methods were the criteria for comparison. The hybrid methods including MST-based [5], GA-based [6], and hierarchical-based [7] *K*-means clustering are three popular hybrid methods for modifying random centroids problems in *K*-means. However, there are other methods which try to improve the *K*-means performance such as principal component analysis [8], different rules for updating the new centroids [9–12] and machine learning online algorithm [13]. Meanwhile, some previous studies report the improvements occurred by *K*-means in other clustering methods [11, 21–24].

To the best of our knowledge, MST-, GA-, and hierarchical-based *K*-means methods utilized in the present study have not been compared in any simulation or experimental study before. Seven datasets used here were different in terms of sample size, numbers of variables, and natural classes. Hence, these three methods compared here from different aspects.

Results of this research indicated that the hybrid methods did not necessarily improve the ordinary *K*-means method; and they even sometimes had poorer performance in some indices than the ordinary *K*-means method (Table 3). The performance of ordinary *K*-means method is improved in hybrid methods only in the number of iterations to reach the final solution. In this regard, hierarchical-based, MST-based, and GA-based clustering methods are in the first to third ranks of convergence rate (Figure 1).

Totally, the hybrid methods could not greatly improve the performance of *K*-means clustering methods in internal validity indices. However, in external validity indices, these methods outperformed the *K*-means clustering method (Table 3).

Finally, since some previous studies reported better performance for these three hybrid methods than the ordinary *K*-means clustering algorithm [5–7] simulation studies are recommended to compare these hybrid methods with *K*-means clustering in terms of initial centroids.

## Figures and Tables

**Table 1:** Description of seven datasets utilized for comparisons among the methods^1^.

Name of dataset	Sample size (+/-)	No. of variables (features)	No. of classes (labels)	No. of optimal clusters^2^
Leukemia	64 (26/38)	4	2	2
Prostate	30 (15/15)	3	2	2
Colon Cancer	111 (56/55)	4	2	2
Haberman	306	3	2	2
Iris	150	4	3	3
Wine	178	13	3	3
Glass	214	10	7	7

^1^Gene Expression Omnibus (https://www.ncbi.nlm.nih.gov/gds) & *University of California, Irvine* Machine Learning Repository (https://archive.ics.uci.edu/ml/datasets.php). ^2^The number of optimal clusters based on the elbow, gap, and silhouette by applying the law of the majority.

**Table 2:** Comparison of four different ordinary clustering methods based on the silhouette and RPT indexes.

Index method	*K*_Means	Hierarchical	DB scan	EM algorithm
Leukemia dataset				
Silhouette	*0.4702*	0.4663	0.2693	0.4419
RPT	*0.880*	0.8612	0.5087	0.8160

Prostate dataset				
Silhouette	0.3265	*0.4029*	0.3339	0.2756
RPT	0.6141	*0.7586*	0.6319	0.5295

Colon				
Silhouette	*0.5248*	0.5189	0.3156	0.5176
RPT	*0.9650*	0.9516	0.5747	0.9478

Haberman				
Silhouette	0.2477	*0.6632*	0.6266	0.1384
RPT	0.4787	*1.21*	1.15	0.2704

Iris				
Silhouette	0.4589	0.4796	*0.5029*	0.3728
RPT	0.8446	0.8614	*0.9085*	0.6812

Wine				
Silhouette	0.2469	0.1575	0.1911	*0.2728*
RPT	0.4788	0.3092	0.3742	*0.5202*

Glass				
Silhouette	0.3411	0.4281	*0.6389*	0.2809
RPT	0.6369	0.7921	*1.17*	0.5148

RPT: robustness performance trade-off.

**Table 3:** Comparison among the hybrid and ordinary *K*-means clustering method based on eleven evaluation criteria.

Indexes	*I*	SSE	Si	RPT	Dunn	RI	ARI	AC	F	HI	VI
Leukemia dataset											
*K*_Means	5	116.2	0.4702	0.880	0.1431	0.8809	0.7617	0.9375	0.8848	0.76197	0.6477
*K*+H	2	116.2	0.4650	0.880	0.1431	0.8809	0.7617	0.9375	0.8848	0.76197	0.6477
*K*+MST	1	116.8	0.4675	0.8719	0.1679	0.9092	0.8183	0.9531	0.9115	0.8184	0.5357
*K*+GA	4	116.2	0.4702	0.8801	0.1431	0.8809	0.7617	0.9375	0.8848	0.76197	0.6477

Prostate dataset											
*K*_Means	6	60.3	0.2677	0.5149	0.0969	0.6298	0.2599	0.7667	0.6247	0.2602	1.51
*K*+H	1	58.1	0.3944	0.6141	0.1549	0.5954	0.1980	0.7337	0.6364	0.2069	1.21
*K*+MST	2	62.1	0.3935	0.7498	0.2239	0.4919	0.0019	0.5667	0.5915	0.0022	1.51
*K*+GA	4	58.7	0.2796	0.5385	0.1498	0.7126	0.4247	0.8333	0.7031	0.4247	1.29

Colon dataset											
*K*_Means	4	161.47	0.5248	0.9650	0.1431	0.8650	0.73	0.9279	0.8638	0.7300	0.7411
*K*+H	2	161.47	0.5248	0.9650	0.1431	0.8650	0.73	0.9279	0.8638	0.7300	0.7411
*K*+MST	3	161.47	0.5248	0.9650	0.1431	0.8650	0.73	0.9279	0.8638	0.7300	0.7411
*K*+GA	2	161.47	0.5248	0.9650	0.1431	0.8650	0.73	0.9279	0.8638	0.7300	0.7411

Haberman dataset											
*K*_Means	6	698.8	0.2477	0.4787	0.023	0.4991	-0.002	0.5196	0.5483	-0.0015	1.83
*K*+H	4	684.4	0.2733	0.5256	0.035	0.5038	0.0083	0.5523	0.5523	0.0085	1.82
*K*+MST	4	702.8	0.3888	0.7427	0.073	0.6189	0.1284	0.7451	0.7270	0.7451	0.1405
*K*+GA	5	682.1	0.2751	0.5305	0.039	0.4997	-0.001	0.5261	0.5488	-0.003	1.83

Iris dataset											
*K*_Means	7	140	0.4589	0.8446	0.02637	0.8322	0.6201	0.8333	0.7452	0.6201	1.079
*K*+H	3	141.1	0.4554	0.8359	0.07756	0.8431	0.6451	0.8533	0.7622	0.6452	1.072
*K*+MST	5	191.7	0.4787	0.8917	0.05309	0.7197	0.4290	0.5732	0.6505	0.4488	1.19
*K*+GA	3	140	0.4589	0.8446	0.02637	0.8322	0.6201	0.8333	0.7452	0.6201	1.079

Wine dataset											
*K*_Means	8	1589.1	0.2469	0.4788	0.1357	0.6915	0.3757	0.6067	0.6237	0.3927	1.42
*K*+H	2	1270.2	0.2905	0.5481	0.2323	0.9543	0.8975	0.9663	0.9319	0.8976	0.39
*K*+MST	4	1270.2	0.2849	0.5481	0.2323	0.9543	0.8975	0.9663	0.9319	0.8976	0.39
*K*+GA	4	1270.2	0.2849	0.5481	0.2323	0.9543	0.8975	0.9663	0.9319	0.8976	0.39

Glass dataset											
*K*_Means	13	687.4	0.3411	0.6369	0.05804	0.6891	0.1966	0.4346	0.4073	0.1966	2.8
*K*+H	2	679.9	0.3458	0.6433	0.04906	0.6926	0.2036	0.4395	0.4116	0.2036	2.73
*K*+MST	4	790.2	0.3021	0.5754	0.06644	0.6531	0.1908	0.3598	0.4327	0.1954	2.60
*K*+GA	10	678.6	0.3427	0.6390	0.04502	0.6879	0.1946	0.4766	0.4062	0.1946	2.84

*I*: number of iteration: ARI: adjusted rand index, −1 < ARI < +1; RI: rand index, RI > 0); VI: variation of information, VI > 0. AC: accuracy, 0 < AC < 1; Si: silhouette, −1 < Si < +1); HI: Huber's *Γ* index, −1 < HI < +1; RPT: robustness-performance trade-off, RPT > 0; *K*+H: hierarchical *K*-means clustering; *K*+MST: minimum spanning tree *K*-means clustering; *K*+GA: genetic *K*-means clustering.

## Data Availability

The data used to support the findings of this study have been deposited in the Gene Expression Omnibus repository for Leukemia, Prostate, and colon cancers (https://www.ncbi.nlm.nih.gov/gds) and in University of California Irvine (UCI) repository for Haberman, Iris, Wine and Glass data sets (https://archive.ics.uci.edu/ml/datasets.php).
